# Protective Role of the *Interleukin 33* rs3939286 Gene Polymorphism in the Development of Subclinical Atherosclerosis in Rheumatoid Arthritis Patients

**DOI:** 10.1371/journal.pone.0143153

**Published:** 2015-11-16

**Authors:** Raquel López-Mejías, Fernanda Genre, Sara Remuzgo-Martínez, Montserrat Robustillo-Villarino, Mercedes García-Bermúdez, Javier Llorca, Alfonso Corrales, Carlos González-Juanatey, Begoña Ubilla, José A. Miranda-Filloy, Verónica Mijares, Trinitario Pina, Ricardo Blanco, Juan J. Alegre-Sancho, Marco A. Ramírez Huaranga, María D. Mínguez Sánchez, Beatriz Tejera Segura, Iván Ferraz-Amaro, Esther Vicente, F. David Carmona, Santos Castañeda, Javier Martín, Miguel A. González-Gay

**Affiliations:** 1 Epidemiology, Genetics and Atherosclerosis Research Group on Systemic Inflammatory Diseases, IDIVAL, Santander, Spain; 2 Rheumatology Department, Hospital Universitario Doctor Peset, Valencia, Spain; 3 Institute of Parasitology and Biomedicine López-Neyra, IPBLN-CSIC, Granada, Spain; 4 Department of Epidemiology and Computational Biology, School of Medicine, University of Cantabria, and CIBER Epidemiología y Salud Pública (CIBERESP), IDIVAL, Santander, Spain; 5 Cardiology Department, Hospital Lucus Augusti, Lugo, Spain; 6 Rheumatology Department, Hospital Lucus Augusti, Lugo, Spain; 7 Rheumatology Department, Hospital General Universitario de Ciudad Real, Ciudad Real, Spain; 8 Rheumatology Department, Hospital Universitario de Canarias, Tenerife, Spain; 9 Rheumatology Department, Hospital Universitario la Princesa, IIS-Princesa, Madrid, Spain; 10 Cardiovascular Pathophysiology and Genomics Research Unit, School of Physiology, Faculty of Health Sciences, University of the Witwatersrand, Johannesburg, South Africa; 11 Health Research Institute of Santiago de Compostela (IDIS), Division of Rheumatology, Clinical University Hospital of Santiago de Compostela, Santiago de Compostela, Spain; Sudbury Regional Hospital, CANADA

## Abstract

**Objectives:**

To determine whether the interleukin-33 (IL-33)-interleukin-1 receptor like 1 (IL-1RL1) signaling pathway is implicated in the risk of subclinical atherosclerosis in patients with rheumatoid arthritis (RA).

**Methods:**

A total of 576 Spanish RA patients from Northern Spain were genotyped for 6 well-known *IL33-IL1RL1* polymorphisms (*IL33* rs3939286, *IL33* rs7025417, *IL33* rs7044343, *IL1RL1* rs2058660, *IL1RL1* rs2310173 and *IL1RL1* rs13015714) by TaqMan genotyping assay. The presence of subclinical atherosclerosis was determined by the assessment of carotid intima-media thickness (cIMT) by carotid ultrasound (US).

**Results:**

RA patients carrying the TT genotype of the *IL33* rs3939286 polymorphism had lower cIMT values than those homozygous for the CC genotype (mean ± standard deviation (SD): 0.71 ± 0.14 mm *versus* 0.76 ± 0.16 mm, respectively) while patients carrying the CT genotype had intermediate cIMT values (mean ± SD: 0.73 ± 0.17 mm). Moreover, RA patients carrying the mutant allele T of the *IL33* rs3939286 polymorphism exhibited significantly lower cIMT values than those carrying the wild allele C (mean ± SD: 0.72 ± 0.16 mm *versus* 0.75 ± 0.18 mm respectively; p = 0.04). The association of both genotype and allele frequencies of *IL33* rs3939286 and cIMT levels remained statistically significant after adjustment for sex, age at the time of US study, follow-up and center (p = 0.006 and p = 0.0023, respectively), evidencing that the potential effect conferred by *IL33* rs3939286 may be independent of confounder factors. No association with other *IL33-IL1RL1* genetic variants was observed.

**Conclusions:**

In conclusion, our results may suggest a potential protective effect of the *IL33* rs3939286 allele T in the risk of subclinical atherosclerosis in patients with RA.

## Introduction

Rheumatoid arthritis (RA) is a complex autoimmune disease associated with progressive disability, systemic complications and early death [1]. Mortality is higher among RA patients than in the general population, and cardiovascular (CV) complications remain a major challenge [2]. The mechanisms leading to accelerated atherosclerosis in RA are complex, including not only the effect of traditional CV risk factors and chronic inflammation [2–3]. In this regard, several pieces of evidence indicate that genetic polymorphisms located inside and outside of the human leukocyte antigen (HLA) region also influence the risk of CV disease in RA [2,4–5]. Subclinical atherosclerosis has been observed in RA patients, even in those without traditional CV risk factors, and abnormally high values of carotid intima-media thickness (cIMT) have been found to predict the risk of CV events in these patients [6].

Interleukin-33 (IL-33) is a newly characterized cytokine that belongs to the IL-1 family [7]. This cytokine is constitutively expressed by tissue barrier cells (such as the epithelial and endothelial cells of many organs), but it is also expressed by some innate immune cells (such as macrophages and dendritic cells). IL-33 displays both nuclear and extracellular effects and mediates its biological function by interacting with its receptor (IL-1 receptor like 1 (IL-1RL1) also called ST2) and coreceptor (IL-1 receptor accessory protein (IL-1RAcP)). Since this binding exerts relevant immunomodulatory functions producing chemokines and pro-inflammatory and Th2-associated cytokines [7], IL-33-IL-1RL1 has been considered as a key pathway involved in inflammatory diseases. In accordance with that, a pathogenic role of IL-33 has been found in RA, where its levels are significantly elevated both in synovial fluid and serum [8]. Moreover, a relationship between baseline detectable IL-33 concentrations and the development of severe subclinical atherosclerosis in patients diagnosed with RA has been described [9].

Regarding genetic studies, polymorphisms located both in *IL33* and *IL1RL1* have been associated with autoimmunity. An association between *IL33* rs3939286 and some immune-mediated diseases such as inflammatory bowel disease (IBD) has been described [10]. Additionally, *IL33* rs7025417 and *IL33* rs4742170, which is in high linkage disequilibrium with *IL33* rs7025417, have been related to coronary artery disease (CAD) and ischemic stroke in non-rheumatic Chinese individuals, respectively [11,12]. Also in the Chinese population, the *IL33* rs7044343 polymorphism has been associated with RA [13]. Regarding *IL1RL1*, a relevant role of *IL1RL1* rs2058660, *IL1RL1* rs2310173 and *IL1RL1* rs13015714 in the development of several inflammatory conditions such as IBD and ankylosing spondylitis has been proposed [10,14].

Taking into account all these considerations, in the present study we aimed to determine, for the first time, whether 6 genetic variants at *IL33* and *IL1RL1*, previously associated with immune-mediated diseases, are involved in the risk of subclinical atherosclerosis in Spanish patients with RA.

## Patients and Methods

### 2.1. Patients and Study Protocol

A set of 576 Spanish patients with RA were included in the present study. Blood samples were obtained from patients recruited from Hospital Lucus Augusti (Lugo) and Hospital Marqués de Valdecilla (Santander) in Northern Spain. A subject’s written consent was obtained according to the declaration of Helsinki, and the study was approved by the Ethics Committees of clinical research of Galicia (CAEI) for Hospital Lucus Augusti in Lugo and Cantabria (CEIC) for Hospital Universitario Marqués de Valdecilla in Santander.

All the patients fulfilled the 2010 classification criteria for RA [15]. In all the cases, patients were assessed for 3 polymorphisms within *IL33* (rs3939286, rs7025417 and rs7044343) and 3 genetic variants located within *IL1RL1* (rs2058660, rs2310173, rs13015714). Also, cIMT was determined by carotid ultrasound (US) technology. Information on the main demographic data, clinical characteristics, CV risk factors and CV events of the patients enrolled in the study is shown in Table 1. Definitions of CV events (ischemic heart disease, heart failure, cerebrovascular accident or peripheral arteriopathy) and those for traditional CV risk factors were established as previously described [2, 6].

**Table 1:** Demographic and clinical characteristics of the Spanish patients with RA included in the study.

Clinical Features	% (n/N)
Patients	576
Main characteristics	
Age at the time of disease onset (years, mean ± SD)	52 ± 14.4
Follow-up (years, mean ± SD)	8.8 ± 7.1
Percentage of women	76.6
Rheumatoid factor positive*	59.6 (335/562)
Anti-CCP antibodies positive	49.9 (252/505)
Shared epitope positive	62.9 (187/297)
Erosions	39.3 (220/560)
Extra-articular manifestations**	21.4 (120/560)
Cardiovascular risk factors	
Hypertension	25.3 (142/561)
Diabetes mellitus	7.1 (40/561)
Dyslipidemia	17.8 (100/561)
Obesity	9.9 (56/561)
Smoking habit	30.6 (172/561)
Patients with cardiovascular events ***	12.8 (74/576)
Ischemic heart disease	4.2 (24/576)
Heart failure	3.8 (22/576)
Cerebrovascular accident	3.3 (19/576)
Peripheral arteriopathy	1.7 (10/576)

RA: Rheumatoid arthritis; SD: Standard deviation; Anti-CCP antibodies: Anti-cyclic citrullinated peptide antibodies.

*At least two determinations were required for analysis of this result.

** Extra-articular manifestations of the disease (if RA patients experienced at least one of the following manifestations: nodular disease, Felty’s syndrome, pulmonary fibrosis, rheumatoid vasculitis, or secondary Sjögren’s syndrome) [2].

***At the time of assessment.

### 2.2. Genotyping

Genomic deoxyribonucleic acid (DNA) from all patients was extracted from peripheral white blood cells using standard procedures.

The selection of single-nucleotide polymorphisms (SNPs) was based on their position with regards to the *IL33-IL1RL1* region and their previously reported associations with several inflammatory diseases. Following these criteria, we performed a tagging using data from HapMap project (http://www.hapmap.org) and Haploview software version 4.2 and considering r^2^>0.8, haplotype frequency >5%, minor allele frequency >10%) (data in S1 and S2 figs). After that, we identified 6 polymorphisms, 3 in the *IL33* region (rs3939286 (C___2762168_10), rs7025417 (C__31940410_20) and rs7044343 (C__29340326_10), located in different haplotype blocks of this locus, and 3 in the *IL1RL1* region (rs2058660 (C__11487892_10), rs2310173 (C___2676437_10) and rs13015714 (C__31439507_10). Although these polymorphisms seem to be apparently non-coding variants, they map and tag both *IL33* and *IL1RL1* regions. Regarding *IL33* region, rs7025417 and rs7044343 are located within *IL33* gene (specifically in the promoter and in the intron 5, respectively) (data in S1 fig). By contrast, rs3939286 is situated in an intergenic region close to *IL33* gene (data in S1 fig). With respect to *IL1RL1* region, whereas rs13015714 is downstream of *IL1RL1* gene, both r2058660 and rs2310173 are placed close to *IL1RL1* gene (data in S2 fig).

All genetic variants were genotyped with TaqMan predesigned single nucleotide polymorphism genotyping assays in a 7900 HT Real-Time polymerase chain reaction (PCR) system, according to the conditions recommended by the manufacturer (Applied Biosystems, Foster City, CA, USA).

Negative controls and duplicate samples were included to check the accuracy of genotyping.

### 2.3. Carotid US Examination

The measurement of the cIMT was performed by carotid US. Patients from Santander were assessed using a commercially available scanner, Mylab 70, Esaote (Genoa, Italy) equipped with 7–12 MHz linear transducer and the automated software guided technique radiofrequency—Quality Intima Media Thickness in real-time (QIMT, Esaote, Maastricht, Holland)—[16]. Patients from Lugo were assessed using high-resolution B-mode ultrasound, Hewlett Packard SONOS 5500, with a 10-MHz linear transducer as previously reported [17]. cIMT was measured at the far wall of the right and left common carotid arteries, 10 mm from the carotid bifurcation, over the proximal 15 mm-long segment. cIMT was determined as the average of three measurements in each common carotid artery. The final cIMT was the largest average cIMT (left or right). Agreement between the two US methods in patients with RA was previously reported [18]. Experts with high experience and close collaboration in the assessment of subclinical atherosclerosis in RA from Lugo and Santander performed the studies.

### 2.4. Statistical Analysis

All genotype data were checked for deviation from Hardy-Weinberg equilibrium (HWE) using http://ihg.gsf.de/cgi-bin/hw/hwa1.pl.

cIMT values were displayed as mean ± standard deviation (SD). The association between genotypes and alleles frequencies of the *IL33* rs3939286, *IL33* rs7025417, *IL33* rs7044343, *IL1RL1* rs2058660, *IL1RL1* rs2310173 and *IL1RL1* rs13015714 polymorphisms and cIMT values was tested using unpaired t test to compare between 2 groups and one-way analysis of variance (ANOVA) to compare among more than two groups. Comparisons of means were adjusted for sex, age at the time of US study, follow-up time and center as potential confounders using analysis of covariance (ANCOVA). Statistical significance was defined as p<0.05. All analyses were performed with STATA statistical software 12/SE (Stata Corp., College Station, TX, USA).

## Results

All genotype distributions were in Hardy-Weinberg equilibrium. The genotyping success was greater than 97% in all the cases.

Results of the comparisons between *IL33* rs3939286, *IL33* rs7025417, *IL33* rs7044343, *IL1RL1* rs2058660, *IL1RL1* rs2310173 and *IL1RL1* rs13015714 according to cIMT values are shown in Table 2. RA patients carrying the TT genotype of the *IL33* rs3939286 polymorphism had lower cIMT values than those homozygous for the CC genotype (mean ± SD: 0.71 ± 0.14 mm *versus* 0.76 ± 0.16 mm, respectively) whereas patients carrying the CT genotype had intermediate cIMT values (mean ± SD: 0.73 ± 0.17 mm) (Table 2). Moreover, RA patients carrying the mutant allele T of the *IL33* rs3939286 polymorphism exhibited significantly lower cIMT values than those carrying the wild allele C (mean ± SD: 0.72 ± 0.16 mm *versus* 0.75 ± 0.18 mm respectively; p = 0.04) (Table 2).

**Table 2:** Association between *IL33-IL1RL1* polymorphisms and cIMT values in RA patients.

*IL33* SNP	Genotype/Allele	cIMT mm	p	P*	*IL1RL1* SNP	Genotype/Allele	cIMT mm	p	P*
		mean ± SD (n)					mean ± SD (n)		
rs3939286	CC	0.76 ± 0.16 (258)	0.10	**0.006**	rs2058660	AA	0.74 ± 0.17 (325)	0.91	0.58
	CT	0.73 ± 0.17 (259)				AG	0.73 ± 0.18 (213)		
	TT	0.71 ± 0.14 (50)				GG	0.75 ± 0.16 (36)		
	C	0.75 ± 0.18 (775)	**0.04**	**0.0023**		A	0.74 ± 0.17 (863)	0.95	0.40
	T	0.72 ± 0.16 (359)				G	0.74 ± 0.18 (285)		
rs7025417	TT	0.75 ± 0.18 (372)	0.15	0.16	rs2310173	GG	0.74 ± 0.17 (154)	0.93	0.76
	TC	0.72 ± 0.17 (172)				GT	0.74 ± 0.18 (280)		
	CC	0.76 ± 0.20 (21)				TT	0.74 ± 0.18 (142)		
	T	0.74 ± 0.18 (916)	0.23	0.16		G	0.74 ± 0.17 (588)	0.77	0.45
	C	0.73 ± 0.17 (214)				T	0.74 ± 0.18 (564)		
rs7044343	TT	0.73 ± 0.17 (280)	0.35	0.33	rs13015714	TT	0.74 ± 0.18 (322)	0.88	0.39
	TC	0.74 ± 0.17 (229)				TG	0.73 ± 0.18 (215)		
	CC	0.77 ± 0.20 (54)				GG	0.74 ± 0.17 (37)		
	T	0.73 ± 0.17 (789)	0.20	0.26		T	0.74 ± 0.18 (859)	0.73	0.29
	C	0.75 ± 0.18 (337)				G	0.73 ± 0.18 (289)		

cIMT: carotid intima-media thickness; RA: Rheumatoid arthritis; SNP: Single nucleotide polymorphism; SD: Standard deviation. Significant results are highlighted on **bold**.

*p-value after adjusting for sex, age at the time of ultrasonography study, follow-up time and center.

Since sex, age at the time of US study, follow-up time and center may act as potential confounders of the results derived from the US assessment; adjustment for these potential confounders was performed by an ANCOVA model. Interestingly, even after adjustment for potential confounders, both genotype and allele frequencies of the *IL33* rs3939286 polymorphism were statistically significant (p = 0.006 and p = 0.0023, respectively) evidencing that the potential effect conferred by *IL33* rs3939286 polymorphism may be independent of confounder factors (Table 2). Nevertheless, no significant association between *IL33* rs7025417, *IL33* rs7044343, *IL1RL1* rs2058660, *IL1RL1* rs2310173 and *IL1RL1* rs13015714 and cIMT values was observed (Table 2).

## Discussion

CV disease is the most common cause of premature mortality in patients with RA being a consequence of accelerated atherosclerosis [1]. In the last years, several studies have been focused on the assessment of biologic mechanisms that influence the risk of CV disease in RA. Accordingly, several genetic markers associated with the risk of subclinical atherosclerosis and CV disease in RA have recently been identified [2,4–5].

Outside the HLA region, cytokine pathway genes, which have critical modulatory effects on innate and adaptive immunity, have been shown to represent a key component of the genetic network associated with immune-mediated processes.

IL-33-IL-1RL1 pathway is a key proinflammatory mediator that may play a pathogenic role in RA [8]. Moreover, detectable IL-33 plasma concentrations at the time of disease diagnosis were found to predict the presence of severe subclinical atherosclerosis in the extended follow-up of patients with RA [9]. Taking into account these considerations and the implication of 6 genetic *IL33-IL1RL1* variants in the susceptibility to several inflammatory diseases [10–14], we aimed to determine the potential association of these genetic polymorphisms with subclinical atherosclerosis in RA. Interestingly, our results suggest a potential protection effect of the mutant *IL33* rs3939286T allele in the risk of subclinical atherosclerosis, established by the assessment of cIMT, in patients with RA. This association remained statistically significant after adjustment for potential confounders, evidencing that this potential effect may be independent of sex, age at the time of the carotid US study, follow-up time and center. It is worth noting that according to the public database RegulomeDB the *IL33* rs3939286 genetic variant appears to be a regulatory DNA element [19]. Consequently, it could be plausible to think that the mutant *IL33* rs3939286T allele might influence the development of subclinical atherosclerosis by regulating the expression of *IL33* and, at last, by decreasing IL-33 levels. Despite having a cohort that might be considered relatively small, our study encompassed the largest series of RA patients with cIMT data ever assessed for genetic studies.

In keeping with our findings, recent studies have described a protective effect of the mutant allele of another *IL33* gene polymorphism, rs7025417, in the risk of CAD in a Chinese population and also in the development of giant cell arteritis in Europeans [11,20]. Since *IL33* rs3939286 and *IL33* rs7025417 genetic variants are located in different haplotype blocks and present a low linkage disequilibrium (LD) (D´ = 0.13 and r^2^ = 0.01), we speculate that either they are independent susceptibility factors for the different phenotypes or they might be tagging the real causal variant.

The results obtained in the present study provide additional evidence on the potential role that genetic factors may play in the development of CV disease in RA. The search for genetic markers associated with CV disease in RA may be important for a better characterization of RA patients at risk of CV disease. They may be useful to predict disease outcome at diagnosis of RA and to establish future therapeutic targets to decrease the risk of CV disease in RA patients.

In conclusion, our results may suggest a potential protective effect of the *IL33* rs3939286 allele T in the risk of subclinical atherosclerosis in patients with RA.

## Supporting Information

S1 Fig Position of the 3 tag *IL33* polymorphisms analyzed in our study. The diamond represents the linkage disequilibrium degree between polymorphisms. The color indicates the D´ (a redder color represents a higher D´). Data was obtained from HapMap project (http://www.hapmap.org) and Haploview software version 4.2 and considering r^2^>0.8, haplotype frequency >5%, minor allele frequency >10%.
(DOCX)

S2 Fig Position of the 3 tag *IL1RL1* polymorphisms analyzed in our study. The diamond represents the linkage disequilibrium degree between polymorphisms. The color indicates the D´ (a redder color represents a higher D´). Data was obtained from HapMap project (http://www.hapmap.org) and Haploview software version 4.2 and considering r^2^>0.8, haplotype frequency >5%, minor allele frequency >10%.
(DOCX)
