# Supplementary figures and images for: Protective Role of the Interleukin 33 rs3939286 Gene Polymorphism in the Development of Subclinical Atherosclerosis in Rheumatoid Arthritis Patients
Source: PLoS One. 2015 Nov 16;10(11):e0143153. doi: 10.1371/journal.pone.0143153 (PMC4646618; doi:10.1371/journal.pone.0143153)

**S1 figure**.


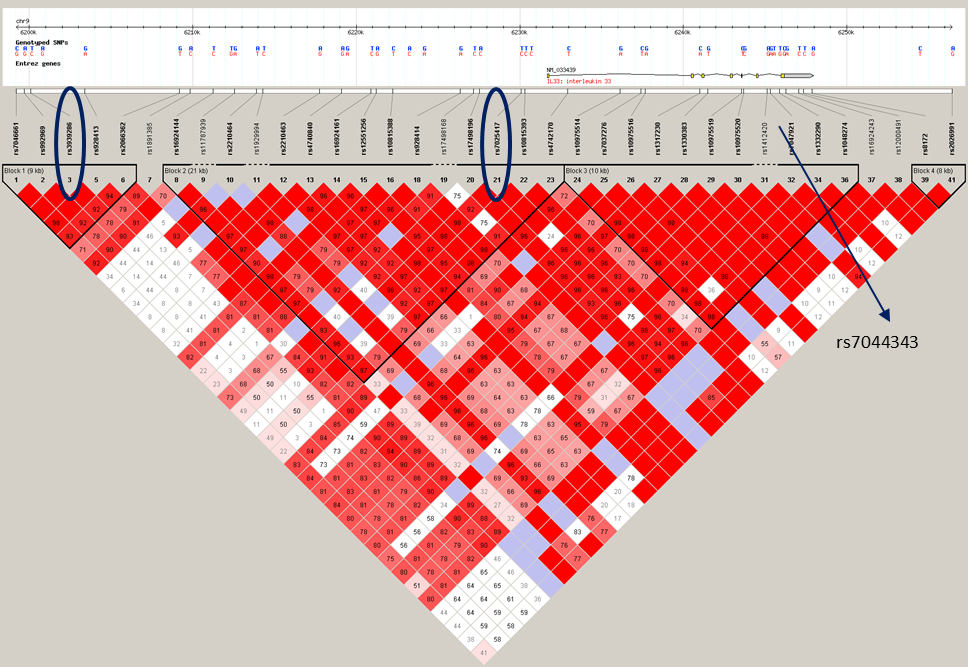

Supplement: S1 Fig — The diamond represents the linkage disequilibrium degree between polymorphisms. The color indicates the D´ (a redder color represents a higher D´). Data was obtained from HapMap project (http://www.hapmap.org) and Haploview software version 4.2 and considering r2>0.8, haplotype frequency >5%, minor allele frequency >10%. (DOCX) [file pone.0143153.s001.docx]

**S2 figure.**


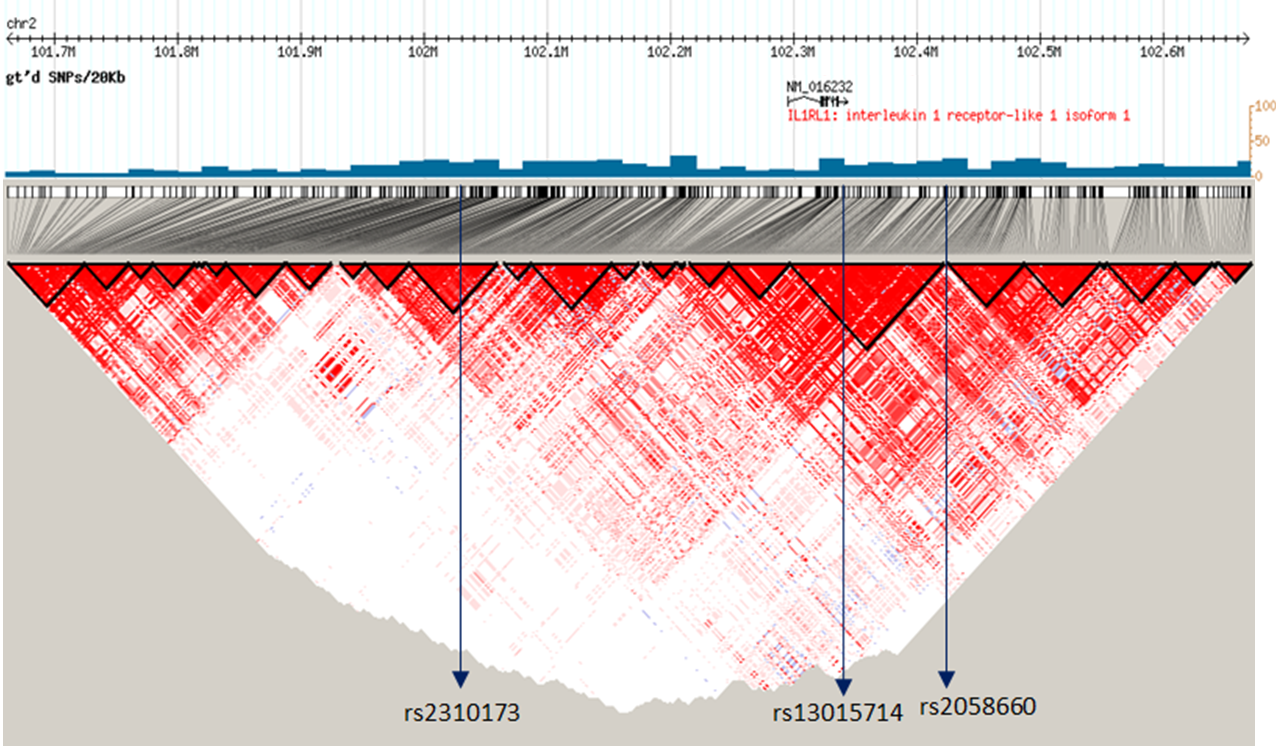

Supplement: S2 Fig — The diamond represents the linkage disequilibrium degree between polymorphisms. The color indicates the D´ (a redder color represents a higher D´). Data was obtained from HapMap project (http://www.hapmap.org) and Haploview software version 4.2 and considering r2>0.8, haplotype frequency >5%, minor allele frequency >10%. (DOCX) [file pone.0143153.s002.docx]
